# An Engineered Synthetic Biologic Protects Against *Clostridium difficile* Infection

**DOI:** 10.3389/fmicb.2018.02080

**Published:** 2018-09-05

**Authors:** Gayatri Vedantam, Joshua Kochanowsky, Jason Lindsey, Michael Mallozzi, Jennifer Lising Roxas, Chelsea Adamson, Farhan Anwar, Andrew Clark, Rachel Claus-Walker, Asad Mansoor, Rebecca McQuade, Ross Calvin Monasky, Shylaja Ramamurthy, Bryan Roxas, V. K. Viswanathan

**Affiliations:** ^1^School of Animal and Comparative Biomedical Sciences, The University of Arizona, Tucson, AZ, United States; ^2^Department of Immunobiology, The University of Arizona, Tucson, AZ, United States; ^3^Bio5 Institute for Collaborative Research, The University of Arizona, Tucson, AZ, United States; ^4^Southern Arizona VA Health Care System, Tucson, AZ, United States

**Keywords:** *Clostridium difficile*, infectious diarrhea, synthetic biology, surface layer protein, *Lactobacillus*

## Abstract

Morbidity and mortality attributed to *Clostridium difficile* infection (CDI) have increased over the past 20 years. Currently, antibiotics are the only US FDA-approved treatment for primary *C. difficile* infection, and these are, ironically, associated with disease relapse and the threat of burgeoning drug resistance. We previously showed that non-toxin virulence factors play key roles in CDI, and that colonization factors are critical for disease. Specifically, a *C. difficile* adhesin, Surface Layer Protein A (SlpA) is a major contributor to host cell attachment. In this work, we engineered Syn-LAB 2.0 and Syn-LAB 2.1, two synthetic biologic agents derived from lactic acid bacteria, to stably and constitutively express a host-cell binding fragment of the *C. difficile* adhesin SlpA on their cell-surface. Both agents harbor conditional suicide plasmids expressing a codon-optimized chimera of the lactic acid bacterium’s cell-wall anchoring surface-protein domain, fused to the conserved, highly adherent, host-cell-binding domain of *C. difficile* SlpA. Both agents also incorporate engineered biocontrol, obviating the need for any antibiotic selection. Syn-LAB 2.0 and Syn-LAB 2.1 possess positive biophysical and *in vivo* properties compared with their parental antecedents in that they robustly and constitutively display the SlpA chimera on their cell surface, potentiate human intestinal epithelial barrier function *in vitro*, are safe, tolerable and palatable to Golden Syrian hamsters and neonatal piglets at high daily doses, and are detectable in animal feces within 24 h of dosing, confirming robust colonization. In combination, the engineered strains also delay (in fixed doses) or prevent (when continuously administered) death of infected hamsters upon challenge with high doses of virulent *C. difficile*. Finally, fixed-dose Syn-LAB ameliorates diarrhea in a non-lethal model of neonatal piglet enteritis. Taken together, our findings suggest that the two synthetic biologics may be effectively employed as non-antibiotic interventions for CDI.

## Introduction

The fastidiously anaerobic, Gram-positive bacillus *Clostridium difficile* is currently the largest contributor to healthcare-associated infections in US hospitals (McDonald et al., 2018). In the United States, an estimated 450,000 cases of *C. difficile* infections (CDI) occur annually (Lessa et al., 2015), costing the healthcare system ≥ $5.4 billion ( Desai et al., 2016). CDI symptoms range from mild/moderate diarrhea, which can progress to serious sequelae including pseudomembranous colitis. Antibiotic suppression of gut flora facilitates colonization by *C. difficile* spores that are commonly present in the environment, which germinate into vegetative cells that produce damaging toxins during late growth phase. Toxigenic (toxin-producing) *C. difficile* strains harbor a 19.6 kb genomic island called the Pathogenicity Locus (PaLoc), that encodes the glucosyltransferase toxins TcdA (309 kDa) and TcdB (267 kDa), which target host-cell G-proteins (Lyras et al., 2009). A third ADP ribosylase toxin CDT (Binary toxin) is present in some strains.

*Clostridium difficile* epidemiology has altered markedly in the past 20 years. Highly virulent strains, associated with severe disease, increased recurrence rate(s) and community onset, have become more prevalent (Ofori et al., 2018). Common human outbreak-associated strains are typed as North American Pulsed-Field type 1 (NAP1) and PCR ribotype 027 (NAP1/027) (Loo et al., 2005). Common veterinary strains (now also isolated from humans) belong to the NAP7/NAP8 clade (Ribotype 078) (Moono et al., 2016). There have been several worldwide outbreaks of NAP1/027/BI CDI since 2002 (Loo et al., 2005).

Bacterial adherence is an important *C. difficile* virulence attribute, with Surface-Layer proteins (SLPs; also known as cell-wall proteins, CWPs) playing key roles. *C. difficile* elaborates up to 29 different SLPs, which are displayed in para-crystalline architecture on the cell surface. *C. difficile* SLPs are also implicated in immune modulation; thus, they are critical non-toxin virulence factors (Bianco et al., 2011; Bruxelle et al., 2016). While SLPs have been proposed as anti-CDI vaccine candidates, many groups (including ours) have reported variability in SLP epitope antigenicity (Biazzo et al., 2013).

Surface-layer protein A (SlpA) and its orthologs are abundant members of the CWP complex in clostridia and lactobacilli. We previously published that pre-incubating human intestinal epithelial cells with *C. difficile* SlpA-enriched preparations, or purified SlpA, or bacteria with anti-SlpA antisera, reduced > 50% *C. difficile* adherence in a dose-dependent manner, implicating SlpA as a major adhesin (Merrigan et al., 2013). Notably, SlpA from a non-toxigenic *C. difficile* isolate blocked adhesion of the strain from which it was derived, as well as a phylogenetically unrelated, non-cognate strain. The degree of adherence inhibition was similar irrespective of the challenge isolate. SlpA is a heterodimer of high-and low-molecular weight (HMW and LMW) subunits. Both subunits bind independently to intestinal cells, with the LMW subunit displaying higher binding efficiency. This guided the engineering of our synthetic strains.

The typical requirement of antibiotics to precipitate CDI, as well as the remarkable efficacy of fecal microbiota transplants in treating refractory CDI, point to one unequivocal conclusion: colonization resistance is an effective and ‘natural’ method to combat CDI (Austin et al., 2014; Terveer et al., 2018). At a practical level, however, fecal transplantation may not be the ideal therapeutic option for all CDI patients (Wang et al., 2016; Gardiner et al., 2018). Alternate approaches that exploit colonization resistance for CDI prevention and/or cure are active areas of investigation (Allen-Vercoe and Petrof, 2013; Petrof and Khoruts, 2014). Probiotics, particularly lactic acid bacteria (LAB), have been considered as safe, palatable options to confer colonization resistance. LAB occupy the same gut niches as *C. difficile* in humans and rodents (Marco et al., 2007), and proliferate to the same or greater extent as *C. difficile* (Chiu et al., 2006). Although LAB can reduce symptoms in some patients, meta-analyses and large-cohort studies suggest variability in LAB protection against CDI (Mergenhagen et al., 2014; Goldenberg et al., 2017). The basis for this variability is unknown, and may reflect inconsistent gut colonization and persistence. Since LAB harbor SLP orthologs and can express heterologous SLP molecules on their surface (Raha et al., 2005; Zhu et al., 2010), we exploited these properties to engineer targeted synthetic biologics with enhanced colonization and immune elicitation properties.

## Materials and Methods

### Cell Lines

The human intestinal epithelial cell line C2_BBe_, a brush border-expressing Caco-2 sub-clone (Peterson and Mooseker, 1992), was used in this study and cultured as previously reported (Roxas et al., 2014).

### Bacterial Strains and Plasmids

All strains and plasmid are described in **Table 1**. LAB were purchased from the American Type Culture Collection (ATCC, Manassas, VA, United States). Specifically, *Lactobacillus casei* strain 334 (Orla-Jensen; Dellaglio et al., 2002), and *Lactobacillus acidophilus* strain 4356 (Roussel et al., 1993) were used for these studies. LAB were grown in De Man, Rogosa and Sharpe (MRS) broth (Duong et al., 2011) and incubated at 30°C in the presence of 5% CO_2_. Bacteria were cultured for 3–5 days to reach saturation [≥1.0 × 10^8^ colony forming units (CFU) per mL].

**Table 1:** Bacterial strains and plasmids used in this study.

Bacteria	Aliases	Species	Genotype	Resistance	Source/Notes
ATCC 393	12473, Orland L-323	*Lactobacillus casei*	Wild type		ATCC
ATCC 4356	Hansen	*Lactobacillus acidophilus*	Wild type		ATCC
DH10B		*Escherichia coli*	F- *mcrA* Δ(*mrr-hsdRMS-mcrBC*) φ80lacZΔM15 *Δlac*X74 *rec*A1 *end*A1 *ara*D139 Δ (*ara, leu*)7697 *galU galK λ- rpsL nupG*/pMON14272/pMON7124		DNA 2.0 (ATUM) Maintenance strain
GC5		*Escherichia coli*	*recA1 endA1 tonA1*		Genesee Scientific
GV1095		*Escherichia coli*	GC5 + pMGM14	Chloramphenicol	This study
GV1096		*Escherichia coli*	GC5 + pMGM12	Chloramphenicol	This study
GV1097		*Escherichia coli*	DH10B + pMGM13	Kanamycin	This study
GV1099		*Lactobacillus casei*	ATCC393 + pTRK848	Erythromycin	This study
GV1100		*Lactobacillus casei*	ATCC393 + pMGM14	Chloramphenicol	This study
GV1101		*Lactobacillus acidophilus*	ATCC4356 + pMGM12	Chloramphenicol	This study
GV1102 (group)		*Lactobacillus acidophilus*	ATCC4356 + pMGM14	Chloramphenicol	This study
Top 10		*Escherichia coli*	F- *mcrA* Δ(*mrr-hsdRMS-mcrBC*) Φ80lacZΔM15 Δ *lacX74 recA1 araD139 Δ(ara leu*)7697 *galU galK rpsL (StrR) endA1 nupG*	Streptomycin	Cloning strain, Thermo Fisher Scientific
6396	Ribotype 012; strain 630	*C. difficile*	Wild type		Gerding Lab, Edward Hines Jr. VA Hospital, Illinois
1470	Ribotype 017	*C. difficile*	Wild type		ATCC
R10079	Ribotype 020	*C. difficile*	Wild type		Cardiff-ECDC^∗^
R20291	Ribotype 027	*C. difficile*	Wild type		Cardiff-ECDC
R26222	Ribotype 078	*C. difficile*	Wild type		Cardiff-ECDC
**Plasmids**					
pTRK848			Expression vector based on a pWV01 origin of replication	Erythromycin	Kok et al., 1984; Duong et al., 2011
pKS1			Broad host-range plasmid pWV01 with a temperature-sensitive *repA* allele	Kanamycin and erythromycin	Shatalin and Neyfakh, 2005
pMGM12			pKS1 + *catP;* self-ligated	Chloramphenicol	This study
pMGM13			pJ241 – DNA 2.0 (now ATUM) maintenance vector harboring the *slpA* chimera fragment	Kanamycin	This study
pMGM14			pMGM12 harboring the *Pme*I-*Fse*I *slpA* chimera-containing fragment from pMGM13	Chloramphenicol	This study


*^∗^Cardiff-European Centre for Disease Prevention and Control [ECDC] C. difficile collection.*

*Lactobacillus acidophilus* and *L. casei* strains ferment the dextrose in MRS to distinct products, and the corresponding pH changes can be detected by including bromophenol blue into the media (MRS-BPB) (Lee and Lee, 2008). *L. acidophilus*, a homo-fermenter, metabolizes dextrose to lactic acid, and the plates remain violet/blue (pH > 4.6); *L. casei*, a hetero-fermenter converts dextrose to acetic acid, and the drop in pH (<3.0) results in a color change to yellow/white. Further, *L. casei*, unlike *L. acidophilus*, can ferment mannitol, and this can be verified by growth on Purple Broth Base (Difco^TM^ Becton, Dickinson and Company Sparks, Glencoe, MD, United States); the acidic change resulting from mannitol fermentation causes the pH indicator bromocresol purple to turn yellow.

**FIGURE 1:**
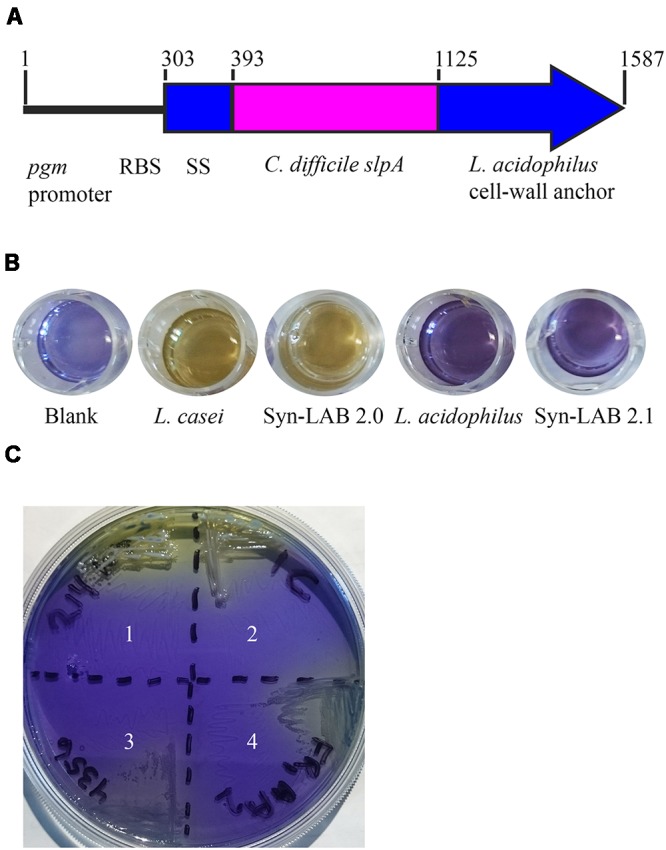
Design and confirmation of Syn-LAB strains. **(A)** Schematic of the *slpA* chimera. Expression is driven by the *L. acidophilus* (LA) *pgm* promoter and ribosome-binding site. The chimera encodes the LA signal sequence (SS), the codon-optimized *C. difficile* extracellular and antigenic SlpA fragment, and the LA Slp cell wall anchor. Syn-LAB strain confirmation via differential growth on mannitol-MRS broth **(B)** and mMRS-BPB agar **(C)**. 1, *L. casei* parent strain; 2, Syn-LAB 2.0; 3, *L. acidophilus* parent strain; 4, Syn-LAB 2.1.

The *slpA C. difficile*/*L. acidophilus* “chimera” fragment (**Figure 1A**) was designed with a strong lactic-acid-bacterial (LAB) promoter [endogenous to the phosphoglycerate mutase (*pgm*) gene in plasmid pTRK848 (Duong et al., 2011)], a lactic-acid-bacterial Shine-Dalgarno (ribosome binding site) sequence (Duong et al., 2011), a signal sequence from a *Lactobacillus acidophilus* S-layer protein, a codon-optimized *C. difficile* strain 630 host-cell-binding fragment, and the *L. acidophilus* SlpA-ortholog cell-wall-binding domain(Michon et al., 2016). The entire fragment (F1) was chemically synthesized (DNA 2.0, now ATUM, Newark, CA, United States), and cloned into the DNA 2.0 maintenance vector pJ241 to yield pMGM13. A second DNA fragment (F2) comprising a broad host-range temperature-sensitive origin of replication (*repA*), and a chloramphenicol resistance gene (*catP*) was also chemically synthesized based on sequence information obtained from the broad host-range plasmid [pKS1, Steen et al., 2010]. This fragment was self-ligated to form pMGM11 (“empty vector”). The two synthesized fragments F1 and F2 harbored *Pme*I and *Fse*I restriction sites at their termini; following digestion with those enzymes, linear F1 and F2 were ligated using T4 DNA ligase (Sigma, St. Louis, MO, United States) to generate pMGM14. Plasmid integrity of pMGM14 was confirmed by complete DNA sequencing. All plasmids were transformed into *Escherichia coli* strains DH10B, TOP10 or NCK1753 for maintenance or propagation purposes, or into *E. coli* GC5 prior to extraction for electroporation. All plasmids were PCR-verified prior to any use in LAB.

### Lactic Acid Bacterial Transformation

The pMGM14 plasmid described above was first extracted from the *E. coli* storage strain, and 10 μg used for each LAB transformation. *Lactobacillus acidophilus* strain 4356 and *Lactobacillus casei* 343 were propagated in Mann-Rogosa-Sharpe (MRS) medium (Remel, Lenexa, KS, United States) per the method of Walker et al. (1996) and Kim et al. (2005). In brief, LAB strains were grown to saturation at 37°C in 5% CO_2_, sub-cultured at a 1:50 dilution, and re-grown using the same process two more times. To shear cell-wall proteins, and prepare the resulting protoplasts for electroporation, a fourth sub-culture was grown for 15 h in MRS supplemented with 1% glycine, and then sub-cultured in the same medium at a 1:50 dilution for an additional 6 h. Bacteria were harvested using centrifugation, and chilled pellets subjected to electroporation with 10 μg plasmid DNA, followed by recovery in MRS broth for 3 h, and growth on MRS-agar + 5 μg/mL chloramphenicol (Sigma, St. Louis, MO, United States) at 30°C in 5% CO_2_. Plasmid was extracted from one-half of a colony of multiple purified transformants, and verified via DNA sequence analysis. The remaining bacterial colonies were propagated in the presence of chloramphenicol at 42°C, the non-permissive temperature, at which *repA* is non-functional, thus selecting for integrants. For pMGM14-based transformants, putative integrants were purified, and assessed for chimeric *slpA* presence by PCR. All confirmations were performed at the non-permissive temperature. Three independently isolated and PCR-verified identical transformants were bio-banked for the *L. casei* strain (herein referred to as Syn-LAB 2.0 clones), and seventeen independently isolated and PCR-verified identical transformants for the *L. acidophilus* strain (herein referred to as Syn-LAB 2.1 clones). For *in vitro* studies only, all LAB strains were propagated at the non-permissive temperature with antibiotic selection as appropriate (5 μg/mL chloramphenicol). However, for all *in vivo* studies, while strains were propagated as above, no selection antibiotic was administered to the animals.

An identical procedure was used to generate empty-vector harboring LAB strains (**Table 1**); these strains were PCR-verified, and used only for *in vivo* studies. Plasmids were maintained episomally; the lack of homology with the LAB host limited the possibility of integration of vector sequences into the chromosome.

### Transepithelial Electrical Resistance (TEER) Measurements

Polarized human intestinal epithelial cells (C2_BBe_; Peterson and Mooseker, 1992) were grown on 0.33-cm^2^ collagen-coated Corning^TM^ Transwells (Thermo Fisher Scientific) for 14 days. Monolayers were treated apically with 1.42 × 10^7^ CFU/well and 1.96 × 10 ^7^ CFU/well of parental and Syn-LAB 2.0 respectively. Measurements were made every hour for 7 h, and at 24 h post-treatment using an epithelial volt-Ohm voltmeter (World Precision Instruments, Sarasota, FL, United States), and TEER calculated by applying Ohm’s Law. An identical setup was used when testing Syn-LAB 2.1 and its *L. acidophilus* parent strain.

### Host Cell Survival Measurement

C2_BBe_ monolayers were treated with media alone, the parent LAB strain, or the corresponding Slp chimera-expressing Syn-LAB derivatives, and host cell viability was assessed using the propidium iodide (PI) uptake assay as described previously (Roxas et al., 2012). Briefly, PI (2 μg/ml; Molecular Probes) was added to the treated cells, and fluorescence measured after 30 min using a microplate reader (Synergy HT; BioTek instruments, Winooski, VT, United States). To estimate maximal PI uptake, a set of wells were treated with 70% methanol prior to PI treatment.

### Immunoblot Analyses

Surface layer proteins (Slp) were extracted from *Lactobacillus* parent and Syn-LAB strain saturated cultures (OD_600nm_= 1.5) using 0.2M glycine (pH2.2), as described by Calabi et al. (2001). For dot blot analyses, 31.25, 62.5, 125, 500 ng, 1 μg, and 2 μg of total protein were blotted on nitrocellulose membranes (Bio-Rad, Richmond, CA, United States). Slp extracts (5–10 μg) were also separated on 4–20% TGX^TM^ pre-cast protein gels (Bio-Rad, Richmond, CA, United States). Separated proteins were transferred to 0.2-μm nitrocellulose membranes (Transblot Cell Apparatus, Bio-Rad). Blots were blocked with 5% non-fat milk in Tris-buffered saline containing Tween 20 (TBST) for 1 h, incubated with antiserum specific to *C. difficile* SlpA (raised against the *C. difficile* strain 630 SlpA (Merrigan et al., 2013) overnight at 4°C and in horseradish peroxidase-conjugated goat anti-rabbit antibody for 1 h at room temperature (Sigma–Aldrich, St. Louis, MO, United States). Membranes were washed five times for 5 min in blocking solution between each incubation step and developed with SuperSignal West Femto Chemiluminescent Substrate (Thermo Fisher Scientific, Rockford, IL, United States).

### Immunofluorescence Microscopy

SlpA chimera expression in *L. casei* WT and Syn-LAB 2.0 and Syn-LAB 2.1 strains was evaluated via immunofluorescence staining using antiserum specific to *C. difficile* SlpA. *Lactobacillus sp* cultures were allowed to settle for 10 min in 12-well plates with poly-L-lysine-coated coverslips. Unattached bacteria were removed, and samples were fixed with 4% paraformaldehyde in PBS (pH 7.4) for 20 min, permeabilized with 0.2% Triton X-100 in PBS for 15 min, quenched with 50 mM NH_4_Cl and 0.125M glycine in PBS for 15 min, and blocked with 5% IgG-free bovine serum albumin (BSA) in PBS for 1 h. Samples were incubated with antiserum specific to *C. difficile* SlpA overnight at 4°C, and then washed three times with 1% IgG-free BSA in PBS. Secondary antibodies (Alexa 488-conjugated goat anti-rabbit IgG antisera; Thermo Fisher Scientific, Waltham, MA, United States) were added at 8 μg/ml in 5% IgG-free BSA for 1 h. Samples were mounted in ProLong Diamond Antifade reagent (Thermo Fisher Scientific, Waltham, MA, United States). Intestinal tissue samples (ileum, cecum, and colon) from LVG Golden Syrian Hamsters (Charles River Laboratories, San Diego, CA, United States) treated with Syn-LAB 2.0 were frozen in OCT embedding medium (Tissue-Tek, Sakura Finetek, Torrance, CA, United States) and stored at -80°C. OCT-mounted tissue samples cut at 3 micron thickness were fixed in 4% paraformaldehyde in PBS (pH 7.4) for 20 min, and processed for SlpA immunofluorescence staining as described above. Samples were stained with 4,6-diamidino-2-phenylindole (DAPI) prior to mounting in ProLong Diamond Antifade reagent. Images were captured using EVOS^®^ FL Imaging System (Thermo Fisher Scientific, Waltham, MA, United States) or DeltaVision Elite Deconvolution Microscope (GE Healthcare, Pittsburgh, PA, United States).

### Flow Cytometry

Parent and transformed *Lactobacillus* sp strains were cultured in MRS broth as described above, and subjected to Gram’s staining to verify purity and morphology. Bacteria were pelleted by centrifugation at 4000 *g* for 2 min. Bacterial pellets were washed gently three times with blocking solution (2% IgG-free BSA in PBS) and then incubated with antiserum specific to *C. difficile* SlpA for 30 min. Secondary antibodies (Alexa Fluor 555-conjugated goat anti-rabbit IgG antisera; Thermo Fisher Scientific, Waltham, MA, United States) were added at 8 μg/ml in 2% IgG-free BSA for 30 min. Samples were washed three times with blocking solution after each antibody incubation step. Stained samples were re-suspended in blocking solution at 10^6^cells/mL density and analyzed via flow cytometry using a BD FACSCANTO II machine (BD Biosciences, San Jose, CA, United States). List mode data files consisting of 10,000 events gated on FSC (forward scatter) vs. SSC (side scatter) were acquired and analyzed using FACSDiva 8.0.1 software (BD Biosciences, San Jose, CA, United States). Appropriate electronic compensation was adjusted by acquiring the cell populations stained with the fluorophore, as well as an unstained control.

### Golden Syrian Hamster Studies

All hamster studies were approved by the Institutional Animal Care and Use Committee of the University of Arizona. The Golden Syrian hamster model was employed to study both colonization/shedding and protection conferred by Syn-LAB strains. For all studies, male hamsters (6–8 weeks; 90–110 g weight) were used.

Shedding studies: Prior to any treatment, hamster stool plated on MRS yielded no colonies, confirming that the animals were devoid of endogenous *Lactobacillus* bacteria. Animals received a daily dose of 10^8^ Syn-LAB 2.0 or 10^8^ Syn-LAB 2.1 respectively. Feeding and enumeration were continued for 6 days. For Syn-LAB 2.0-treated animals only, oral clindamycin (prescription solution; clindamycin sulfate; University of Arizona Pharmacy; 30 mg/kg) was administered on Day 4, and LAB detection monitored until Day 6. Fecal pellets were collected daily, and pellets were re-suspended in PBS, homogenized, serially diluted and plated on the appropriate Syn-LAB selective medium containing chloramphenicol. Colonies were detected only in stool samples from Syn-LAB-treated animals, and not from untreated controls. Shedding from all animals was statistically indistinguishable. For added confirmation, select colonies were 16S PCR-verified for *L. casei* as well as the presence of the chimeric *slpA*.

Challenge studies: These were performed in two modalities, a “fixed-dose” and a “continuous dose” format. All animals received clindamycin (prescription solution; clindamycin sulfate; University of Arizona Pharmacy; 30 mg/kg). 1000 spores of *C. difficile* strain CD630 was used in the challenge studies where indicated, and 10^8^ CFU LAB was used wherever indicated. Group 1 animals received clindamycin on day -3 but no other intervention (black line in **Figure 8**). Group 2 hamsters received clindamycin (day -3) and *C. difficile* challenge (day 0), but no LAB treatment (blue line in **Figure 8**). Group 3 hamsters received *L. casei* parent strain/empty vector on days -6, -5, -4, -2, -1, and 0, and clindamycin on day -3, followed by *C. difficile* challenge on day 0 (magenta line in **Figure 8**). Group 4 hamsters received Syn-LAB 2.0 on days -6, -5, -4, -2, -1, and 0, clindamycin on day -3, and *C. difficile* challenge on day 0 (green line in **Figure 8**).

For continuous-dose studies, the clindamycin dose and timing was similar to that above, and only the “*C. difficile*,” “Empty Vector” and “LAB” groups as above were evaluated. Both “Empty Vector” and Syn-LAB 2.0 (“LAB”) were continuously dosed at 10^8^ CFU per animal per day starting at Day -6 before infection, until death/euthanasia.

Where appropriate, infections commenced 72 h post-antibiotic administration, and the challenge strain used was *C. difficile* strain 630 (1000 spores; orally administered in PBS). Animals were monitored for disease symptoms (wet-tail, ruffled coat, lethargy) through the course of the studies. Moribund hamsters or those meeting the criteria for euthanasia were administered 270 mg/kg commercial euthanizing solution (Euthanasia III, MedPharma Inc, Pomona, CA, United States). Euthanized hamsters were dissected for visualization of gross pathology, and cecal contents harvested and plated on selective medium for recovery and molecular typing of *C*. *difficile* (using 16s-23s rDNA intergenic fragment profiling and comparison with the organisms used for infection). In all studies, and all groups, fecal pellets were also collected daily, re-suspended in PBS, homogenized, serially diluted and plated on *C. difficile* or *L. casei* selective medium as appropriate.

Immune response studies: for these experiments, age- and weight-matched Golden Syrian hamsters (at least 3 per group) were administered 10^8^ CFU Syn-LAB 2.0 daily, or left untreated, for 21 days. Animals were then euthanized, whole blood harvested via cardiac puncture, and serum immediately retrieved after blood was centrifuged at 1000 *g* for 10 min. This material was aliquoted and stored at -80°C until further use. For immune response assessments, the same methodology as immunoblotting above was used, but serum from Syn-LAB or mock-treated animals was used as the source of primary antibody.

### Neonatal Piglet Studies

All piglet studies were approved by the Institutional Animal Care and Use Committee of the University of Arizona; we assessed Syn-LAB 2.1 safety, tolerability and efficacy in protecting against *C. difficile* challenge. Newborn male and female piglets were obtained via assisted delivery from a local antibiotic-free, small-volume farm, and transferred to the University of Arizona Central Animal Facility within 2 h of birth. On Day 2 post-birth, piglets were treated with oral vancomycin (50 mg/kg; prescription solution, University of Arizona Pharmacy) to ablate any pre-existing *C. difficile* colonization. On day 6 post-birth, piglets were administered 10^10^ Syn-LAB 2.1 in milk replacer every 8 h. On day 7, a subset of animals was administered a non-lethal dose of 1000 *C. difficile* spores of strain 630. Monitoring included checks every 8 h thereafter, with weight, stimulus response and dehydrations scores recorded. Upon completion of the study, piglets were anesthetized with Ketamine/Xylazine, and then humanely euthanized with commercial euthanizing solution (Euthanasia III, MedPharma Inc, Pomona, CA, United States) followed by cardiac puncture. Histologic analyses included standard hematoxylin-eosin staining of colonic tissues following standard methodologies (Kiernan, 2008), and immunofluorescence staining of tissues with anti-*C. difficile* SlpA serum as described in detail above for visualization of Syn-LAB 2.0.

### Statistical Analysis

Multiple statistical tests were employed and utilized the Excel-Stat application to determine significance for experiments involving quantitation. For growth and bacterial burden, Student’s *t-*tests were performed to compute differences between parental and Syn-LAB strains, and errors bars calculated from standard deviation(s). For *in vivo* studies, Kaplan–Meier survival curves were computed followed by Log-Rank tests for *post hoc* analyses.

## Results

### Construction of *C. difficile*/Lactic Acid Bacterium (LAB) SlpA Chimera-Encoding Plasmids and Generation of Syn-LAB Strains

A 7740-base pair (bp) shuttle vector was assembled from chemically synthesized fragments. The vector has the following features: a temperature-sensitive *repA* allele and a chimeric *slpA* (**Figure 1A**) that includes; (1) a strong *L. acidophilus* (LA) promoter [upstream sequences of the phosphoglycerate mutase (*pgm*) gene (base pairs 178339-178600 of the *L. acidophilus* NCFM genome, Genbank accession number CP000033.3 (Altermann et al., 2005; Duong et al., 2011) (2) an LA Shine-Dalgarno (ribosome binding site) sequence (CCTGCA); and (3) sequences encoding a chimeric SlpA that includes a LA signal sequence (amino acids 1–30 of LA SlpA) (Altermann et al., 2005), codon-optimized *C. difficile* strain 630 LMW SlpA host-cell-binding region (amino acids 1–243) (Karjalainen et al., 2002), and the LA cell-wall-binding domain (amino acids 291–444) (Altermann et al., 2005). Precise engineering of the entire 7740 base pair vector was confirmed by complete DNA sequencing.

**FIGURE 2:**
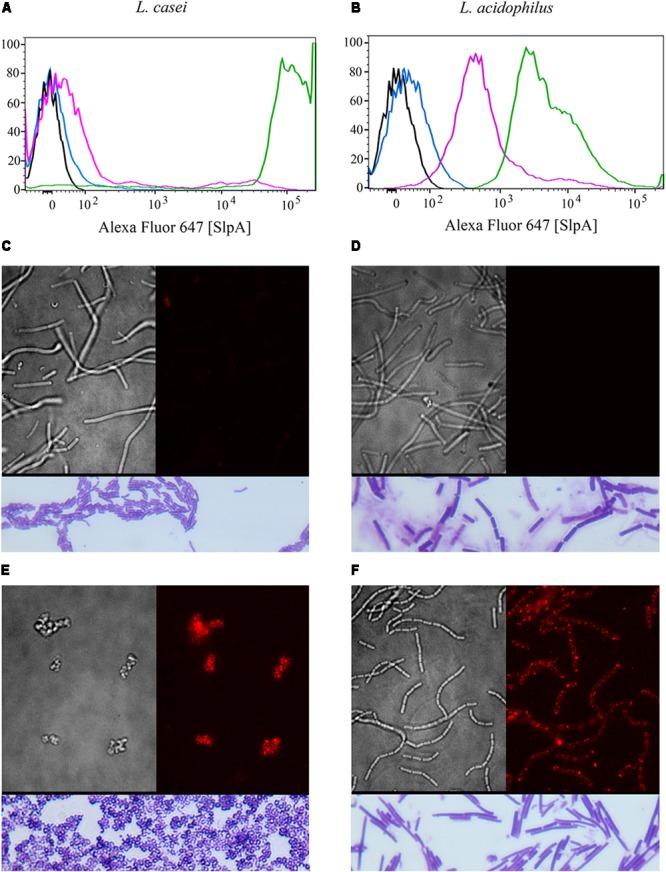
Syn-LAB 2.0 and Syn-LAB 2.1 surface-display chimeric SlpA. **(A,B)** Fluorescence-activating cell sorting analyses. **(A)** Black, unstained *L. casei* parent strain; Blue, unstained Syn-LAB 2.0; Magenta, SlpA-stained *L. casei* parent strain; Green, SlpA-stained Syn-LAB 2.0 (median fluorescence > 100,000). *L. casei* does not have a classic S-layer, therefore, Syn-LAB shift is unique. **(B)** Black = unstained *L. acidophilus* parent strain; Blue, unstained Syn-LAB 2.1; Magenta, SlpA-stained *L. acidophilus* parent strain; Green, SlpA-stained Syn-LAB 2.1 (median fluorescence > 4,000). *L. acidophilus* has a native S-layer, therefore, chimeric SlpA is detected as a discrete, strong fluorescence shift. **(C–F)** microscopy; **(C)** brightfield image, *L. casei* parent strain with minimal detectable SlpA fluorescence; **(D)** brightfield image, *L. acidophilus* parent strain with undetectable SlpA fluorescence; **(E)** immunofluorescence, Syn-LAB 2.0 with intense, punctate, SlpA staining, and **(F)** immunofluorescence, Syn-LAB 2.1 with intense SlpA staining. All strains were probed with a *C. difficile* -specific anti-SlpA serum. Images are representative of at least 20 fields and > 1000 bacteria visualized. All images were visualized with a high-resolution DeltaVision deconvolution microscope. Gram’s stained bacteria are shown in rectangles below each of **(C–F)**.

Lactic acid bacterial strains were individually transformed with this shuttle vector. All transformants were recovered at the permissive temperature (30°C) where RepA is functional, confirmed by biochemical tests and selective plating (**Figures 1B,C**), and further confirmed by multiple PCR tests (not shown). Finally, transformants were propagated with selection at the non-permissive temperature (37–42°C) that allows for recovery of integrants. A total of 3 independently isolated *Lactobacillus casei* transformants were obtained (herein collectively referred to as Syn-LAB 2.0 clones). Seventeen independently isolated *Lactobacillus acidophilus* transformants were also obtained (herein referred to as Syn-LAB 2.1 clones). Syn-LAB clones of each LAB species were confirmed via PCR, phenotypic (growth; not shown) and biochemical tests (**Figures 1B,C**), and bio-banked. The unique carbohydrate fermentation profiles were exploited to readily distinguish between the two species: *L. casei* can ferment mannitol, and converts dextrose to acetic acid, and the corresponding media acidification manifested as a color change to yellow/white in the presence of appropriate pH indicators. *L. acidophilus*, on the other hand, does not ferment mannitol, and converts dextrose to lactic acid; the media remained purple/blue in the presence of the corresponding carbohydrates. Gram’s staining revealed that in contrast to the rod-shaped morphology of the parent *L. casei* strain, Syn-LAB 2.0 cells were shorter and curved (**Figures 2C,E**). Syn-LAB 2.1 bacteria were indistinguishable from the parent *L. acidophilus* strain (**Figures 2D,F**).

One isolate each of Syn-LAB 2.0 and Syn-LAB 2.1 as well as the respective parent *Lactobacillus* sp strains were used with appropriate antibiotic selection for the *in vitro* studies presented below. For *in vivo* studies, Syn-LAB 2.0, Syn-LAB 2.1, a combination thereof, or “empty vector” harboring *Lactobacillus* sp. strains were tested; no selection antibiotics were used in animals.

### Syn-LAB Strains Display Chimeric *C. difficile* SlpA

SlpA chimera expression was confirmed via multiple methodologies for both engineered biologics (Syn-LAB 2.0 and Syn-LAB 2.1). First, flow cytometry was used to determine the degree of heterologous (chimeric) SlpA surface display. In actively growing cultures, almost 100% of Syn-LAB 2.0 bacteria displayed the *C. difficile* SlpA chimera (confirmed by fluorescence shifts in the engineered isolate compared to the parent strains (**Figure 2A**). Similar results were obtained for Syn-LAB 2.1 (**Figure 2B**), confirming robust SlpA display in that biologic as well.

Second, chimeric SlpA expression and display was visualized via immunofluorescence using anti-*C. difficile* SlpA antiserum (Merrigan et al., 2013). In contrast to the parent *L. casei* strain (**Figure 2C**), the engineered Syn-LAB 2.0 derivative revealed dense SlpA staining (**Figure 2E**). Similarly, Syn-LAB 2.1 (**Figure 2F**), but not the parent *L. acidophilus* strain (**Figure 2D**), exhibited intense and punctate surface SlpA staining. Staining was specific since no signal was detected on either Syn-LAB strain with pre-immune serum (not shown).

### Chimeric *C. difficile* SlpA Is Incorporated Into the Lactic Acid Bacterial Cell Surface

**FIGURE 3:**
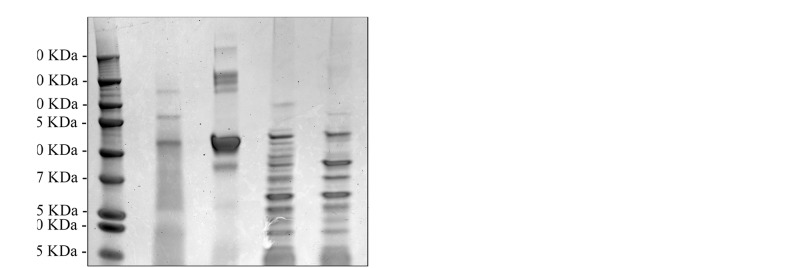
Chimeric *C. difficile* SlpA is incorporated into the lactic acid bacterial (LAB) cell wall. **(A)** Sheared total S-Layer Proteins from the *L. casei* parent strain (Lc) or Syn-LAB 2.0; and the *L. acidophilus* parent strain (La), or Syn-LAB 2.1. **(B)** Immunoblot probing the total Surface Layer Proteins of parent and Syn-LAB strains with antiserum specific to *C. difficile* SlpA. The red arrow points to unique, expected-size bands present only in Syn-LAB 2.0 and Syn-LAB 2.1 respectively. The dotted line indicates that the original immunoblot was edited to remove irrelevant lanes.

We also confirmed that the *C. difficile*-specific SlpA visualized in **Figure 2** above was due to incorporation of the chimeric protein into the LAB cell wall. Sheared total Surface-Layer (S-layer) proteins from Syn-LAB 2.0 and Syn-LAB 2.1, and the isogenic parent strains (Merrigan et al., 2013), were separated via agarose gel electrophoresis (**Figure 3A**), and immunoblotted using the anti-SlpA antiserum. Both synthetic biologics, but not the isogenic parent strains, displayed altered total S-layer profiles, and a discrete unique band, appropriate to the expected size of the chimera, (**Figure 3B**). Mass spectrometry confirmed the presence of chimeric SlpA sequences (not shown). Due to the polyclonal nature of the antiserum other non-specifically reacting bands were also observed in the parent strains.

### Chimeric SlpA Expression Preserves Epithelial Barrier Function

**FIGURE 4:**
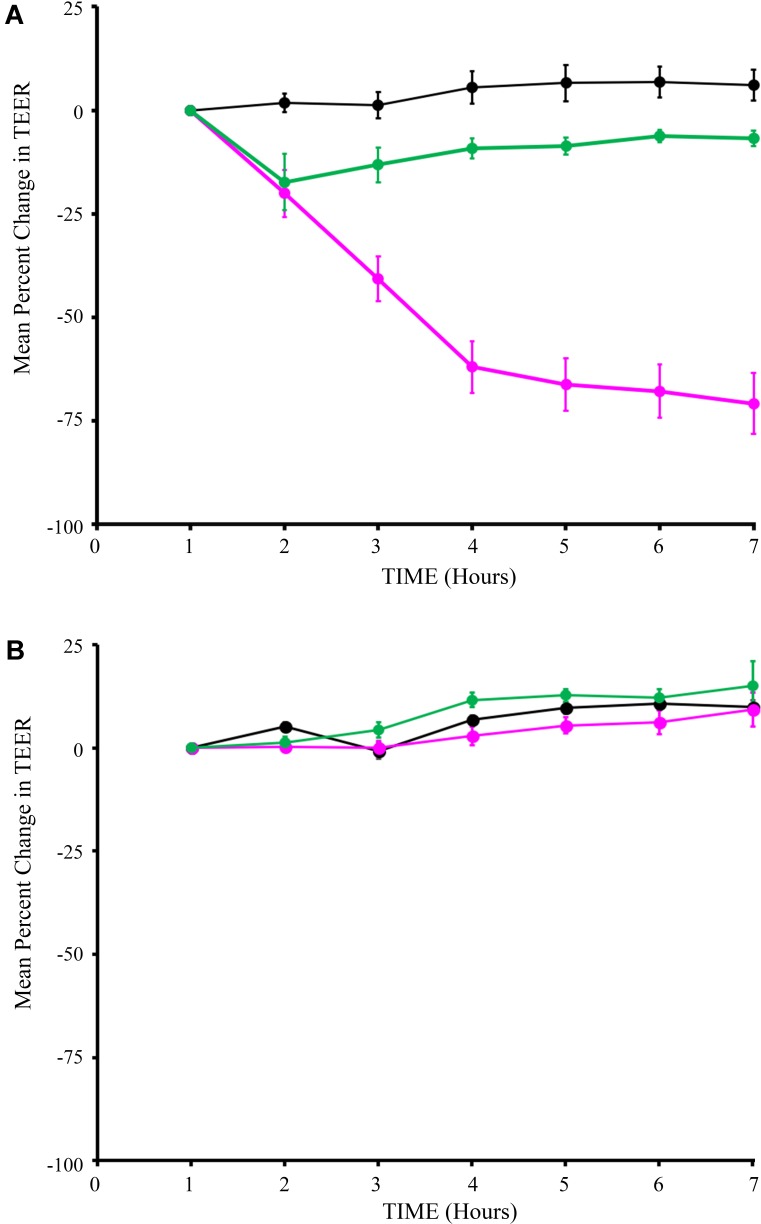
Syn-LAB 2.0 preserves epithelial barrier function. Transepithelial electrical resistance (TEER) measurements of C2_BBe_ monolayers that were mock-treated (black line), or exposed to the parent *L. casei* strain (green line), or Syn-LAB 2.0 (magenta line; **A**), or parent *L. acidophilus* strain (green line), or Syn-LAB 2.1 (magenta line; **B**), for 8 h. Experiments were performed in at least three biological replicates. Host cells were treated with 100 bacteria per cell (MOI = 100).

We performed a series of studies to rule out potential adverse effects of the engineered strains on intestinal epithelial cell health and function. First, potential impact of Syn-LAB strains on intestinal epithelial barrier function was assessed via *trans*-epithelial electrical resistance (TEER) measurements. Unexpectedly, the parent *L. casei* strain decreased TEER over a 7-h period, with changes becoming consistently apparent as early as 3 h post-application (**Figure 4A**). In contrast, addition of Syn-LAB 2.0 did not significantly alter TEER relative to mock-treated cells. Similarly, Syn-LAB 2.1, or the parent *L. acidophilus* strain, had no impact on the TEER of C_2BBe_ cells (**Figure 4B**). This suggests that Syn-LAB 2.0 and Syn-LAB2.1 do not disrupt host epithelial barrier function.

### Chimeric SlpA Display Does Not Compromise Host Cell Viability

**FIGURE 5:**
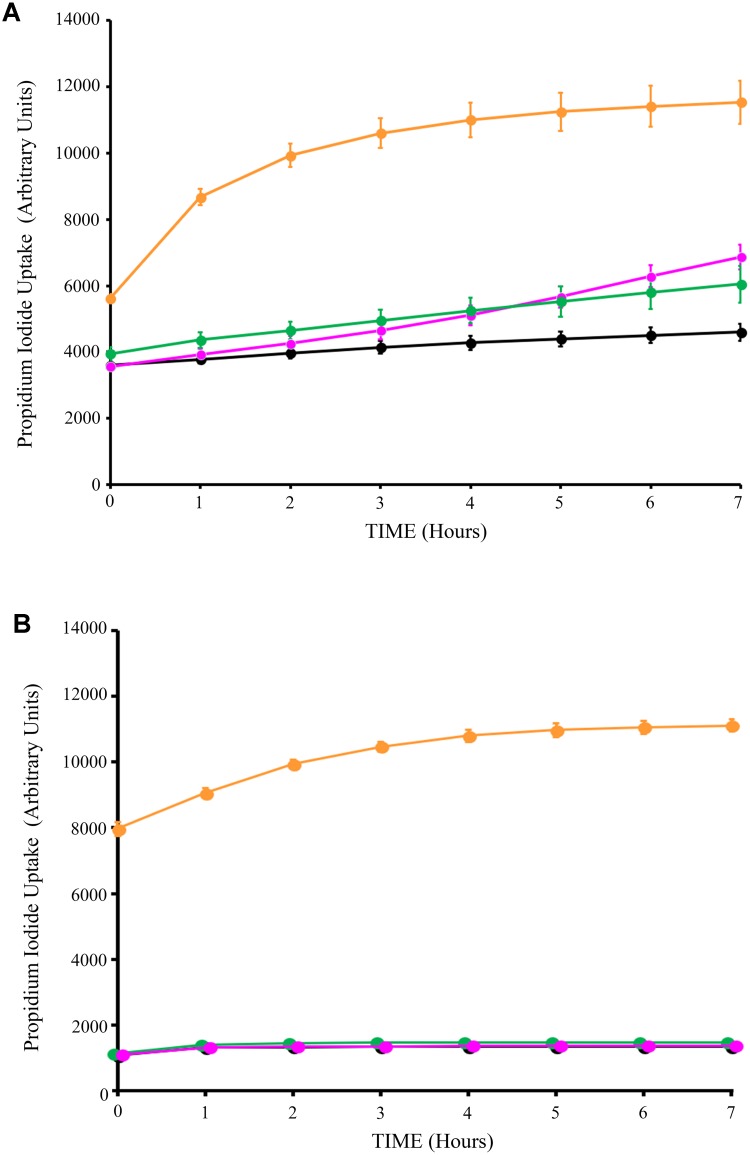
Lactic acid bacteria (LAB) treatment does not impact host-cell viability. Propidium iodide uptake assays of C2_BBe_ monolayers mock-treated (black line), or exposed to the parent *L. casei* strain (green line), or Syn-LAB 2.0 (magenta line; **A**), or parent *L. acidophilus* strain (green line), or Syn-LAB 2.1 (magenta line; **B**), for 8 h. Orange line shows methanol-treated monolayers representative of maximal cell death. Experiments were performed in at least three biological replicates. Host cells were treated with 100 bacteria per cell (MOI = 100).

Next, we assessed the impact of Syn-LAB on the survival of host intestinal epithelial cells. C2_BBe_ cells were grown to confluence, and either mock-treated, or treated with *L. casei, L. acidophilus*, or the respective Syn-LAB derivatives for up to 8 h. Cell death was continuously monitored via propidium iodide (PI) uptake, a DNA-intercalating dye that only enters dying cells with compromised cell membranes. *L. casei, L. acidophilus*, and the respective chimera-expressing Syn-LAB derivatives did not significantly impact epithelial cell viability relative to mock-treated cells (**Figures 5A,B**).

### Syn-LABs Are Safe and Tolerable in Multiple Animal Models, and Robustly Colonize the Mammalian Gastrointestinal Tract (GIT)

PCR- and microbiologically verified pure cultures of Syn-LAB strain 2.0 was used for these studies, and prepared for *in vivo* dosing at ∼10^8^ bacteria per 200 μL volume of PBS. To conserve animals, the shedding/colonization assessments were performed as pilot studies. For broad assessment of Syn-LAB shedding, two animal models (Golden Syrian hamsters and neonatal piglets) were utilized, with each model testing one of the two Syn-LAB strains respectively.

**FIGURE 6:**
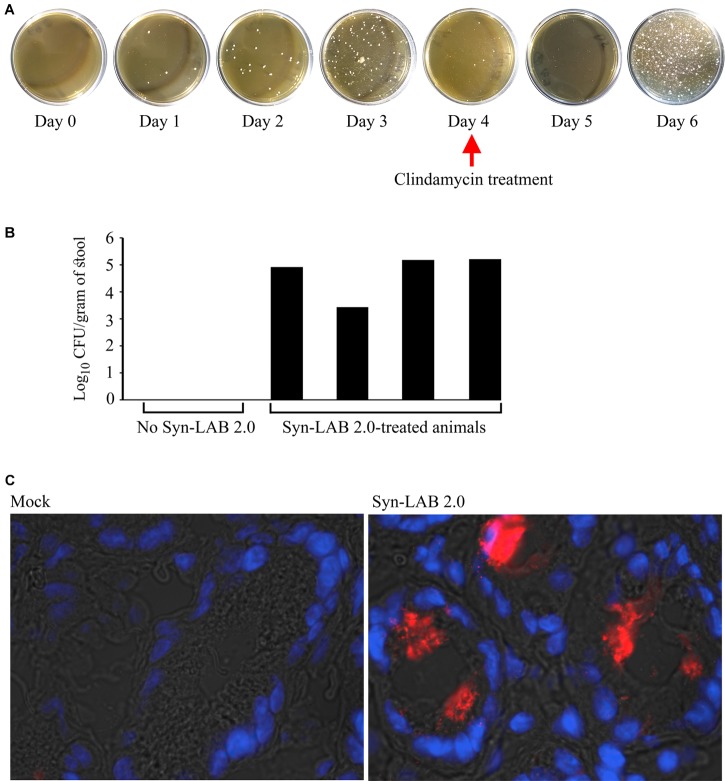
Syn-LAB colonization of Golden Syrian Hamsters. **(A)** Syn-LAB 2.0 colonizes the hamster gastrointestinal tract, and is shed as early as 1 day post-administration. Hamster stool pellets from animals continuously administered Syn-LAB 2.0 at 10^8^CFU/day cultured on MRS-chloramphenicol. Antibiotic treatment on Day 4 ablates Syn-LAB 2.0 shedding only for 1 day, followed by continued detection from Day 5 onward. Data are representative of 8 infected animals. **(B)** Syn-LAB 2.0 shedding is comparable in all treated animals (black bars), but not detectable in control, untreated, animals. **(C)** immunofluorescence analysis using anti-*C. difficile* SlpA serum reveals intense staining in the colonic lumen of Syn-LAB 2.0-fed animals (right), but not mock-treated animals (left). Images are representative at least 10 fields visualized per section.

Golden Syrian hamsters received either no treatment (control), or a single dose of the broad-spectrum antibiotic clindamycin, followed by 6 daily doses of the *L. casei*-based Syn-LAB 2.0 (antibiotic “pre-treatment” group), or 3 days of Syn-LAB 2.0, followed by a single dose of oral clindamycin (as above), followed by another 3 days of Syn-LAB 2.0 (antibiotic “mid-cycle treatment” group). Syn-LAB treated hamsters started shedding the biologic on Day 1 post-administration; this continued until the end of the study (similar studies were performed for Syn-LAB 2.1, with identical observations; not shown). Hamsters that received clindamycin on Day 4 post-Syn-LAB showed no evidence of the biologic on Day 5, confirming the *in vivo* susceptibility of Syn-LAB 2.0 to standard antimicrobial therapy (**Figure 6A**). However, when Syn-LAB administration was restarted, shedding resumed at a magnitude similar to that observed prior to antimicrobial therapy (**Figure 6A**). Importantly, all hamsters were similarly colonized, with Syn-LAB fecal titers reaching, or exceeding, 10^5^ colony forming units/gram stool on Day 3 post-treatment (**Figure 6B**). Immunofluorescence studies of colonic tissues harvested post-necropsy revealed dense luminal staining only from Syn-LAB 2.0 treated hamsters (**Figure 6C**, right panel) as compared with mock-treated animals (**Figure 6C**, left panel), confirming *C. difficile* SlpA expression in the hamster gastrointestinal tract. Finally, Syn-LAB 2.0 was avidly consumed by all hamsters, with no requirement for a pre- or post-dosing sweetened electrolyte “chaser.” Safety and tolerability were also confirmed via lack of any adverse effects in any Syn-LAB-treated hamsters, as well as appropriate activity and alertness throughout the study.

### Continuous Syn-LAB Administration Induces an Anti-*C. difficile* SlpA Immune Response

**FIGURE 7:**
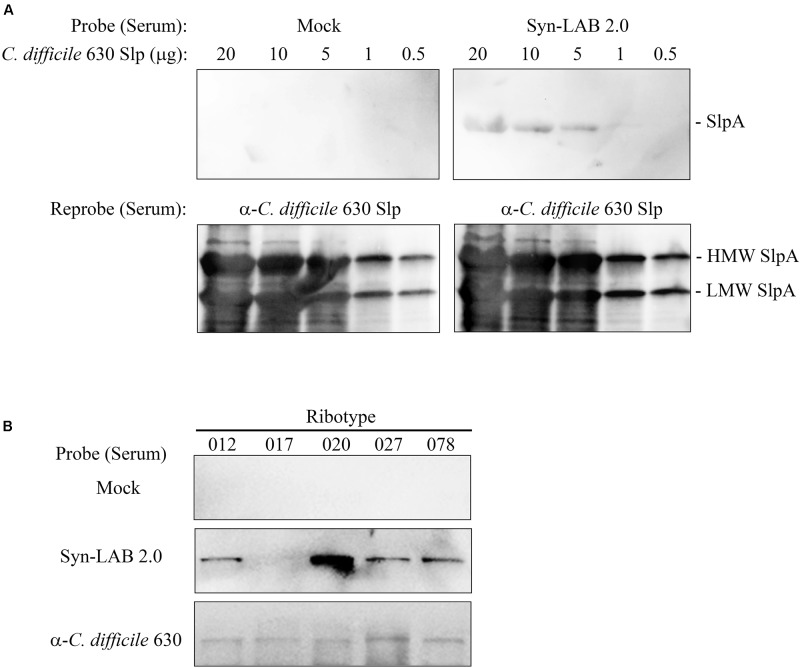
Syn-LAB 2.0 elicits an anti-*C. difficile* SlpA immune response. **(A)** Top panels, dose-response immunoblots of S-layer proteins (20 to 0.5 μg) from *C. difficile* strain 630 probed with serum from a Syn-LAB 2.0-treated animal (right), or serum from an untreated, age- and weight-matched hamster (left). Bottom panels, to verify efficient separation and transfer of the S-layer proteins, the membranes were stripped and re-probed with a polyclonal *C. difficile* anti-SlpA antiserum. Both *C. difficile* SlpA subunits were detected (arrows). **(B)** S-layer proteins from *C. difficile* clinical isolates of diverse ribotypes probed with serum from an untreated animal (upper row), a Syn-LAB 2.0-treated animal (middle row), or with anti-*C. difficile* strain 630 SlpA serum (bottom row).

While the primary goal was to design biologic agents that could competitively occupy *C. difficile* attachment sites in the gut, we also explored the possibility of an anti-SlpA immune response following long-term Syn-LAB administration. Golden Syrian hamsters were continuously administered Syn-LAB 2.0 as a once-daily 10^8^ CFU dose for 55 days. Hamsters shed the biologic consistently throughout the process confirming that they were appropriately colonized. Age- and weight-matched control hamsters received no treatment. At the end of the study, hamsters were humanely euthanized, whole blood collected, and immunoblot-based analyses performed to assess anti-Syn-LAB immune response. Serum from Syn-LAB 2.0-treated hamsters (**Figure 7A**, top right panel), but not from mock-treated animals (**Figure 7A**, top left panel), detected *C. difficile* strain 630 SlpA in a dose-dependent manner. Presence of Slp proteins in the corresponding membranes was verified by re-probing the blots with a SlpA-specific antiserum previously generated in our laboratory (**Figure 7A**, lower panels). Finally, the same experiments were performed using Slp preparations from clinically-relevant isolates of diverse *C. difficile* ribotypes (012, 017, 020, 027, 078). Reactivity was observed only when serum from Syn-LAB-treated hamsters was used (**Figure 7B**). This suggested that the Syn-LAB SlpA moiety elicited a cross-reactive immune response (recognition of non-cognate *C. difficile* SlpA).

### Syn-LABs Protect Syrian Golden Hamsters From *C. difficile*-Induced Death

Since single-species probiotics are thought to have limited ability to protect against CDI (Wullt et al., 2003; Vernaya et al., 2017), we used a combination of Syn-LAB 2.0 and Syn-LAB 2.1 in hamster protection studies. A mixed culture of the biologics (10^8^ CFU total) was administered to antibiotic-sensitized Golden Syrian hamsters either as a fixed dose (FD) formulation (6 doses) or as a continuous dose (CD) formulation (3 days prior to clindamycin until the end of the study). Syn-LAB-treated hamsters were compared to those receiving *C. difficile* alone, or those administered LAB containing the empty vector. Challenge studies used a high inoculum (∼1000 spores) of *C. difficile* strain 630 [a virulent, outbreak-associated isolate (Merrigan et al., 2013)].

**FIGURE 8:**
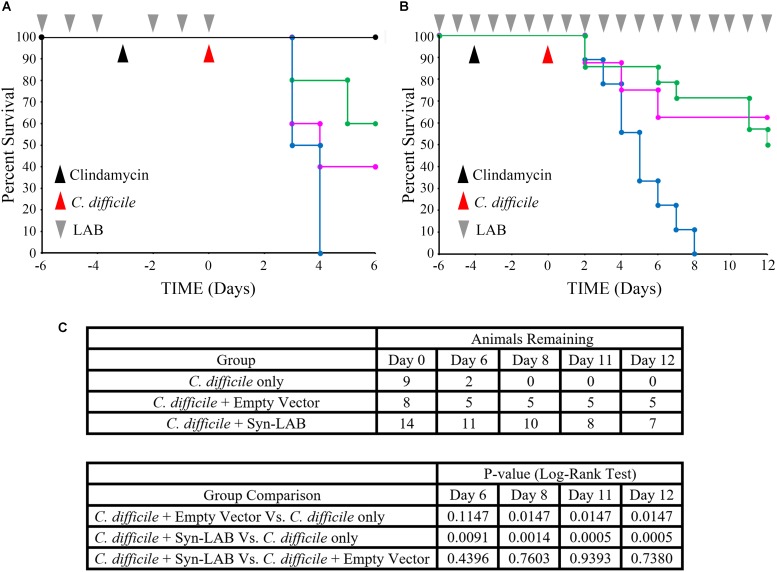
Syn-LAB biologics protect hamsters from lethal CDI. **(A,B)** Co-administration of Syn-LAB 2.0 and 2.1 protects Golden Syrian hamsters from CDI. **(A)**, pilot study; Kaplan–Meier survival plot. Pre-treatment with Syn-LABs (6 once-daily doses of 1 × 10^10^ CFU each) delays death of hamsters infected with 1000 *C. difficile* spores. Black line, untreated, uninfected animals; blue line, *C. difficile*-infected animals; magenta line, animals treated with parent LAB strains harboring empty vector, and then infected; green line, animals treated with Syn-Lab 2.0+2.1 and then infected. **(B)**, powered study, Kaplan–Meier survival plot. Continuous Syn-LAB 2.0+2.1 dosing (pre- and post-infection) prevents death of *C. difficile*-infected hamsters. blue line, *C. difficile*-infected animals; magenta line = animals treated with parent LAB strains harboring empty vector, and then infected; green line = animals treated with Syn-Lab 2.0+2.1 and then infected. **(C)** Group comparisons and statistical test results. Number of surviving animals in powered study (top), and Log-Rank tests (bottom). *p* ≤ 0.01 = significant.

Fixed dosing of the Syn-LAB combination significantly delayed death of hamsters compared to mock-treated animals, as well as those administered the empty-vector-harboring strains (**Figure 8A**). Continuous administration of Syn-LABs afforded statistically significant protection against CDI throughout the course of 12 days of infection (**Figure 8B**). Specifically, and as compared with untreated hamsters, protection was highly significant at multiple time points during the infection course (*p* = 0.0091, *p* = 0.0014, *p* = 0.0005, *p* = 0.0005 at 6, 8, 11 and 12 days post-infection respectively). This was in contrast to the protection afforded when hamsters were administered the parent LAB strain harboring the empty vector (*p* = 0.1147, *p* = 0.0147, *p* = 0.0147, *p* = 0.0147 on Days 6, 8, 11, and 12 post-infection respectively; 99% confidence interval for significance). Additionally, parent strain-treated hamsters succumbed to disease earlier in the infectious course, and were more often found moribund, with symptoms consistent with fulminant CDI (profound wet-tail, lethargy, sternal recumbency and cecal hemorrhage).

Re-administration of clindamycin to Syn-LAB-treated, *C. difficile-*challenged hamsters 14 days post infection did not result in disease or mortality (not shown). This suggested that Syn-LAB-mediated colonization resistance also ablated *C. difficile* persistence. Taken together, Syn-LAB administration was highly protective in the hamster model described above.

### Fixed-Dose Syn-LAB Administration Protects Neonatal Piglets From *C. difficile*-Induced Diarrhea

Preliminary neonatal piglet studies used healthy, newborn animals treated with vancomycin on Day 4 post-farrowing to ensure elimination of any carryover *C. difficile* bacteria from the farm. Control piglets were given PBS followed by *C. difficile* challenge, whereas treated piglets were administered three 10^8^ CFU doses of the fast-growing Syn-LAB 2.1 strain over 24 h. Syn-LAB 2.1 was detected as early as 24 h after the first dose, and shedding continued until the end of the study (not shown).

**FIGURE 9:**
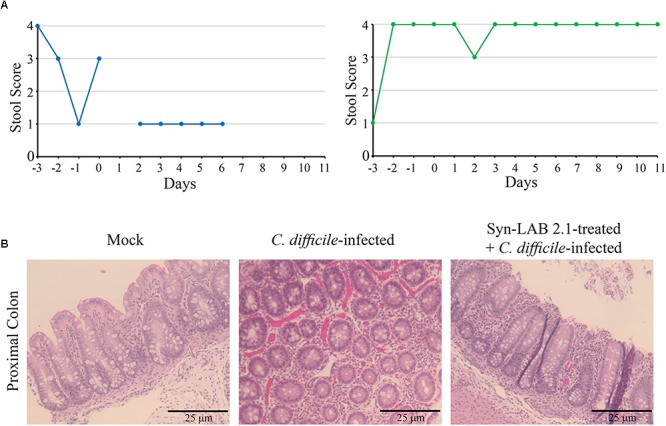
Syn-LAB 2.1 protects piglets from CDI diarrhea. Pilot, non-lethal CDI model. **(A)** Stool consistency scoring; 1 = diarrheic, 4 = fully formed. Left, *C. difficile*-infected animal, with consistently low stool scores indicating unremitting diarrhea. Right, Syn-LAB 2.1 treated + *C. difficile*-infected animal, with consistently high stool score, indicating no diarrhea. The example shown is representative of 3 animals studied. The animal in the left panel was euthanized after the last time-point shown due to unremitting diarrhea, increasing dehydration and inappetence. Similar results for the other two animals. All infected animals in the group received 1000 spores of *C. difficile*. **(B)** microscopic examination of proximal colon tissues from piglets. Left, hematoxylin-eosin staining of tissue from uninfected animal showing normal epithelium, little/no inflammatory infiltrate and no overt damage or necrosis. Middle, *C. difficile*-infected animal, revealing gross hemorrhage with an abundance of inflammatory infiltrates. Right, Syn-LAB 2.1-treated and *C. difficile-*infected animal tissue showing marked reduction in both overt hemorrhage and inflammation.

In this model, CDI [1000 spores for piglets (Steele et al., 2010)], resulted in profuse diarrhea (**Figure 9A**, left panel, stool score of 1). Diarrheic symptoms in these piglets continued unabated for at least 3 days, at which time the accumulated dehydration and inappetance criteria necessitated euthanasia. Microscopic examination of colonic tissues from infected piglets revealed gross hemorrhage with an abundance of inflammatory infiltrates (**Figure 9B**, middle panel). However, piglets that received a 1-day administration of Syn-LAB 2.1 had well-formed stool (Stool score of 3–4; **Figure 9A**, right panel), as well as normal activity and appetite. Colonic tissue from these animals showed markedly less hemorrhage and inflammatory damage compared to those from piglets with CDI alone (**Figure 9B**, right panel).

## Discussion

With the emergence of outbreak-associated strains in the past decade, CDI has become a problem of considerable magnitude in terms of human and economic costs (Viswanathan et al., 2010). The protection offered by a healthy microbiota, known as colonization resistance, is the most effective foil against CDI (Seekatz et al., 2018). Although antibiotic-mediated dysbiosis is the most typical precipitating factor for CDI, traditional therapeutic options rely on administration of more antibiotics (McDonald et al., 2018). Apart from concerns of increased antibiotic resistance in *C. difficile*, this approach aggravates intestinal dysbiosis and, in a subset of infected individuals, results in recurrent disease (Viswanathan et al., 2010). Therefore, strategies that exploit colonization resistance to prevent or treat CDI can not only be effective in mitigating disease, but also address the underlying dysbiosis.

Two broad therapeutic approaches that exploit colonization resistance are fecal microbiota transplantation (FMT) and probiotic administration. In randomized trials, FMT efficacy ranges from ∼50% to 90% based on delivery and number of infusions (van Nood et al., 2013; Youngster et al., 2014; Cammarota et al., 2015; Kelly et al., 2016; Lee et al., 2016), but this procedure is logistically challenging and could pose undefined risks to patients (Wang et al., 2016; Gardiner et al., 2018); as such, it is recommended only for patients that repeatedly fail antibiotic therapy (at least 3 CDI episodes; IDSA-SHEA guidelines (McDonald et al., 2018). While probiotics are more palatable and pose fewer risks, they show variable efficacy in treating CDI (Rezaie and Pimentel, 2014; McFarland, 2015; Barker et al., 2017; Goldenberg et al., 2017; Alberda et al., 2018). Some studies have shown probiotic efficacy when used in patients with no CDI history, but differences in formulation, dose, dosing duration and species composition preclude strong conclusions being drawn in favor of probiotics as CDI interventions. Indeed, the latest IDSA-SHEA recommendations for CDI intervention do not mention probiotics as a treatment option, and no recommendation is made for the agents in primary disease prevention (McDonald et al., 2018). Our goal was to develop a biologic agent for colonization resistance against CDI with consistent and robust efficacy against CDI, but with a safety profile comparable to extensively used probiotics. We, therefore, sought to engineer the “Generally Regarded as Safe” (GRAS) organisms *Lactobacillus casei* and *Lactobacillus acidophilus* to express *C. difficile* surface adhesins and, thereby, competitively exclude the pathogen from intestinal surfaces.

Lactobacilli can be extraordinarily recalcitrant to manipulation; this is both an advantage and a liability. The difficulties encountered in introducing or extracting DNA from the various species portends well for the use of the organisms in probiotic preparations due to reduced risk of horizontal acquisition of antibiotic resistance genes from endogenous microbiota. However, laboratory manipulation poses unique challenges. Indeed, only electroporation with high amounts (≥10 μg) of DNA, and a strain-specific optimized protocol, is the recommended method to transform *Lactobacillus* sp; despite this, reported efficiencies may be as low as 1 transformant/μg DNA (Vogel and Ehrmann, 1996). On balance, however, *Lactobacillus casei* and *Lactobacillus acidophilus* offer unique advantages that can be exploited for CDI treatment. *L. casei* can suppress the inflammatory cytokines produced in response to CDI (Boonma et al., 2014), upregulate mucin gene expression (Mattar et al., 2002), and also appears to confer human subjects some protection from CDI when administered as a fermented drink (Wong et al., 2014; Alberda et al., 2018). *L. acidophilus* has been shown to decrease *C. difficile* toxin gene expression and also protect animals in a murine CDI model (Yun et al., 2014). However, data regarding the strain-specific benefits of these organisms, or their consistently beneficial use in diverse patient cohorts, are scarce. For Syn-LAB engineering, we used pure, genome-sequenced, antibiotic sensitive strains of both genera. Lactobacilli are notoriously recalcitrant to taxonomic classification, and phenotypic or biochemical identification, and therefore need to be subjected to extensive molecular identification to confirm species purity (Pal et al., 2012). The Syn-LAB 2.0 antecedent strain is morphologically and biochemically distinguishable from the Syn-LAB 2.1 parent, allowing for clear discrimination of the strains.

The CD/LAB chimeric SlpA was robustly expressed in both *Lactobacillus* species, integrated into the cell wall, and displayed on the surface; thus, the surface display of the chimera was not hindered by presence of the native LAB S-layer. We assessed Syn-LAB dosing and maintenance, and its ability to protect against a high dose of *C. difficile* spore infection (> 1000 spores) in the lethal hamster model of infection [a spore dose of ∼100 causes 100% lethality (Sattar et al., 2015)]. Continuous Syn-LAB dosing was easily achieved in hamsters, and the bacteria were readily detected in high numbers in the stool as early as 1 day post-administration. With fixed dosing, however, the biologics gradually declined in numbers in the stool, and were below the level of detection within 5 days post-administration (not shown). Such tunable maintenance of the biologics in the gut would be sparing of the endogenous microbiota, whose reestablishment after antibiotic insult is critical to CDI relief.

Even with fixed, pre-challenge dosing (6 doses), the engineered Syn-LAB 2.0 strain afforded protection against CDI. With continuous dosing of the biologic agent, however, there was significant protection against disease as well as mortality, even in the absence of any plasmid maintenance antibiotics (none were used in our animal studies). Treatment with the parent LAB strains was partially protective with continuous dosing compared to no treatment at all; this was not statistically significant compared to untreated animals, and was more variable, correlating with observations in human clinical studies where probiotic use does not consistently protect against CDI (Mills et al., 2018).

Beyond colonization resistance, the elicitation of anti-*C. difficile* SlpA antibody response in Syn-LAB-treated hamsters is noteworthy. In previous studies, recombinant SlpA-vaccinated mice exhibited a modest decrease in subsequent fecal *C. difficile* shedding, and an anti-SlpA response afforded partial protection against CDI (Biazzo et al., 2013; Bruxelle et al., 2016). Thus, Syn-LAB strains could afford long-term protection from new- or re-infection with diverse *C. difficile* strains in the community or healthcare setting. The use of these targeted biologics, therefore, via a once-daily oral administration as we tested herein, is likely to be suitable for multiple hosts.

Unlike the lethal hamster model, piglets are natural hosts to CDI, and display symptoms similar to human infections. Recent studies have highlighted CDI burden in agriculture, and its impact on the swine industry (Grzeskowiak et al., 2016; Stein et al., 2017; Kim et al., 2018). Our preliminary studies in this model are promising: as with the hamster studies, Syn-LAB was delivered and maintained in the piglets with ease, with the biologic being shed in the stool consistently. Even with a single-day FD regimen, the piglets were protected against CDI-induced diarrhea, in contrast to animals given *C. difficile* alone.

## Conclusion

Individuals (or animals) who are not appropriate candidates for anti-CDI immunization, those anticipating extended-duration antibiotic treatment, or those in long-term care facilities (LTCFs) where the risk of acquiring CDI is high may benefit from Syn-LAB-type agents. Further, asymptomatic carriage of *C. difficile* is considered to be a major factor in pre-disposing patients to active CDI (Blixt et al., 2017; Caroff et al., 2017), and Syn-LAB administration may substantially reduce this risk.

## Limitations of this Study

The studies as presented support Syn-LAB biologics as powerful new tools to prevent CDI in multiple mammalian systems. However, we recognize limitations that will need to be addressed by fully powered studies prior to completion of Syn-LAB pre-clinical testing. First, Syn-LAB efficacy will need to be tested against an even wider panel of recent *C. difficile* clinical isolates than those shown in **Figure 7**. However, the strong serum reactivity from Syn-LAB-treated animals against diverse, clinically relevant *C. difficile* ribotypes is encouraging. Second, Syn-LAB 2.0 and 2.1 may differ in their individual propensities to protect against CDI, and this needs to be investigated further. Third, we need to evaluate if the anti-SlpA antibody response results in protective immunity against *C. difficile* challenge, and parse this from colonization resistance provided by the biologic itself. Finally, the ability of Syn-LABs as a therapeutic (and not just a prophylactic as presented herein) remains to be tested – and this will involve efforts to determine biologic efficacy at different time-points post-infection. These limitations notwithstanding, our studies show strong evidence that the engineered Syn-LAB strains have considerable potential as primary or adjunct therapeutic agents against CDIs in multiple mammalian systems.

## Author Contributions

GV and VV conceptualized and funded these studies, wrote the manuscript, and provided full project oversight. JL, JK, MM, and JLR designed, performed, optimized, and interpreted the experiments. CA, FA, AC, RC-W, AM, RCM, RM, and SR performed, supported and fully participated in all animal studies as well as some *in vitro* confirmation studies (FA and RM). All the authors read the manuscript and provided feedback.

## Conflict of Interest Statement

The authors declare that the research was conducted in the absence of any commercial or financial relationships that could be construed as a potential conflict of interest.
